# Chemical Genetic Inhibition of Mps1 in Stable Human Cell Lines Reveals Novel Aspects of Mps1 Function in Mitosis

**DOI:** 10.1371/journal.pone.0010251

**Published:** 2010-04-22

**Authors:** Tale Sliedrecht, Chao Zhang, Kevan M. Shokat, Geert J. P. L. Kops

**Affiliations:** 1 Department of Physiological Chemistry, Cancer Genomics Centre, University Medical Center (UMC) Utrecht, Utrecht, The Netherlands; 2 Netherlands Proteomics Centre, Utrecht, The Netherlands; 3 Department of Cellular and Molecular Pharmacology, University of California San Francisco, San Francisco, California, United States of America; Virginia Tech, United States of America

## Abstract

**Background:**

Proper execution of chromosome segregation relies on tight control of attachment of chromosomes to spindle microtubules. This is monitored by the mitotic checkpoint that allows chromosome segregation only when all chromosomes are stably attached. Proper functioning of the attachment and checkpoint processes is thus important to prevent chromosomal instability. Both processes rely on the mitotic kinase Mps1.

**Principal Finding:**

We present here two cell lines in which endogenous Mps1 has been stably replaced with a mutant kinase (Mps1-as) that is specifically inhibited by bulky PP1 analogs. Mps1 inhibition in these cell lines is highly penetrant and reversible. Timed inhibition during bipolar spindle assembly shows that Mps1 is critical for attachment error-correction and confirms its role in Aurora B regulation. We furthermore show that Mps1 has multiple controls over mitotic checkpoint activity. Mps1 inhibition precludes Mad1 localization to unattached kinetochores but also accelerates mitosis. This acceleration correlates with absence of detectable mitotic checkpoint complex after Mps1 inhibition. Finally, we show that short-term inhibition of Mps1 catalytic activity is sufficient to kill cells.

**Conclusions/Significance:**

Mps1 is involved in the regulation of multiple key processes that ensure correct chromosome segregation and is a promising target for inhibition in anti-cancer strategies. We report here two cell lines that allow specific and highly penetrant inhibition of Mps1 in a reproducible manner through the use of chemical genetics. Using these cell lines we confirm previously suggested roles for Mps1 activity in mitosis, present evidence for novel functions and examine cell viability after short and prolonged Mps1 inhibition. These cell lines present the best cellular model system to date for investigations into Mps1 biology and the effects of penetrance and duration of Mps1 inhibition on cell viability.

## Introduction

To maintain a stable genome, cells have evolved a variety of processes that ensure accurate chromosome segregation. In early mitosis, kinetochores of sister chromatids attach to microtubules emanating from opposite spindle poles. Correct end-on attachment of microtubules to kinetochores relies on the error-correction machinery that destabilizes improper attachments through the actions of the Aurora B kinase [1]. As long as unattached kinetochores persist, the onset of anaphase is prevented by a surveillance mechanism called the mitotic checkpoint that will halt cell cycle progression until all chromosomes are stably attached to the mitotic spindle [2]. The mitotic checkpoint will be satisfied upon stable biorientation of all chromosomes, after which chromosome segregation is allowed to proceed. Proper execution of chromosome biorientation and mitotic checkpoint signaling relies on a set of multifunctional kinases, one of which is the dual specificity kinase Mps1 [3].

First discovered to regulate spindle pole body duplication in budding yeast [4], Mps1 was subsequently found to additionally regulate the mitotic checkpoint [5] and spindle assembly [6]. Regulation of the mitotic checkpoint by Mps1 is evolutionary conserved and has been shown in fission yeast, fruit flies, *Xenopus* egg extracts and human cells [7]–[11]. Mps1 exerts this control, at least in part, through regulating kinetochore localization of several checkpoint proteins including Mad1 and Mad2 [9], [11], [12]. Recently, Mps1 was also reported to regulate sister chromatid biorientation in both budding yeast and humans [12], [13]. In human cells, Mps1 promotes biorientation by regulating Aurora B activity through phosphorylation of the chromosomal passenger complex (CPC) member Borealin [12], [14].

Due to its central role in mitosis, misregulation of Mps1 kinase activity results in chromosomal instability (CIN) and subsequent aneuploidy, a hallmark shared by cells from solid tumors [15], [16]. Inefficient activation of Mps1 results in weakened mitotic checkpoint activity and the persistence of falsely attached chromosomes, causing frequent but non-lethal chromosome segregation errors [16]. Conversely, reduction of Mps1 activity has recently been shown to sensitize tumor cells but not normal cells to low doses of taxol by elevating the frequency of chromosome missegregations to near-lethal levels [17]. Partial inhibition of Mps1 might therefore be an effective anti-cancer therapy.

Although RNAi studies have uncovered several aspects of human Mps1 biology, the multifunctional character of Mps1 has prevented detailed and temporally controlled investigations into the different roles Mps1 might play in mitosis. Inhibition using the small molecules SP600125 and cincreasin has proved to be useful [18], [19], but cincreasin does not inhibit Mps1 in human cells [19] and the non-specific nature of SP600125 makes it an unfavorable choice to study Mps1. A more controlled approach is the use of chemical genetics, in which endogenous kinase is replaced by an engineered protein containing a mutated gatekeeper residue [20]. These gatekeeper mutants render the kinase specifically sensitive to inhibition by non-hydrolysable bulky ATP analogs such as chemically modified variants of the Src inhibitor PP1. This approach has previously been described for Mps1 in budding yeast [6], [13] and in human cells in combination with transient RNAi [21]. The use of transient RNAi, however, introduces uncertainties regarding efficiency of knock down and reproducibility.

We present two cell lines in which endogenous Mps1 has been stably replaced with gatekeeper mutants. In-depth analysis of these clonal cell lines showed that addition of bulky PP1 analogs allowed rapid and reversible inhibition of Mps1 kinase activity in a highly penetrant and reproducible manner. Using these cell lines we dissected the different roles of Mps1 in mitosis, further establishing known functions in checkpoint regulation and error-correction and providing novel insights in the function of Mps1 catalytic activity in checkpoint regulation.

## Results

### Mps1 is selectively inhibitable in two engineered cell lines

To study the role of Mps1 in different mitotic processes, we created cell lines in which Mps1 kinase activity could specifically and reversibly be inhibited. For this, we engineered the ATP-binding pocket of Mps1 to create a kinase with unique preference for bulky ATP-like small molecules [20]. As previously shown for Mps1 in a transient expression system [21], Mps1^M602A^ signifies such an analog sensitive (as) version. In addition to M602A, we also tested M602G, which similarly enlarges the ATP-binding pocket of Mps1 and may provide stronger inhibition [6]. To ensure that mutation of the gatekeeper residue did not affect Mps1 functionality, the ability of both Mps1^M602A^ and Mps1^M602G^ to sustain an active checkpoint in Mps1 RNAi cells was investigated. Similar to LAP-Mps^WT^, both LAP-Mps1^M602A^ and LAP-Mps1^M602G^ reconstituted checkpoint signaling in response to nocodazole when endogenous Mps1 was transiently replaced with these proteins (Fig. S1A).

**Figure 1:**
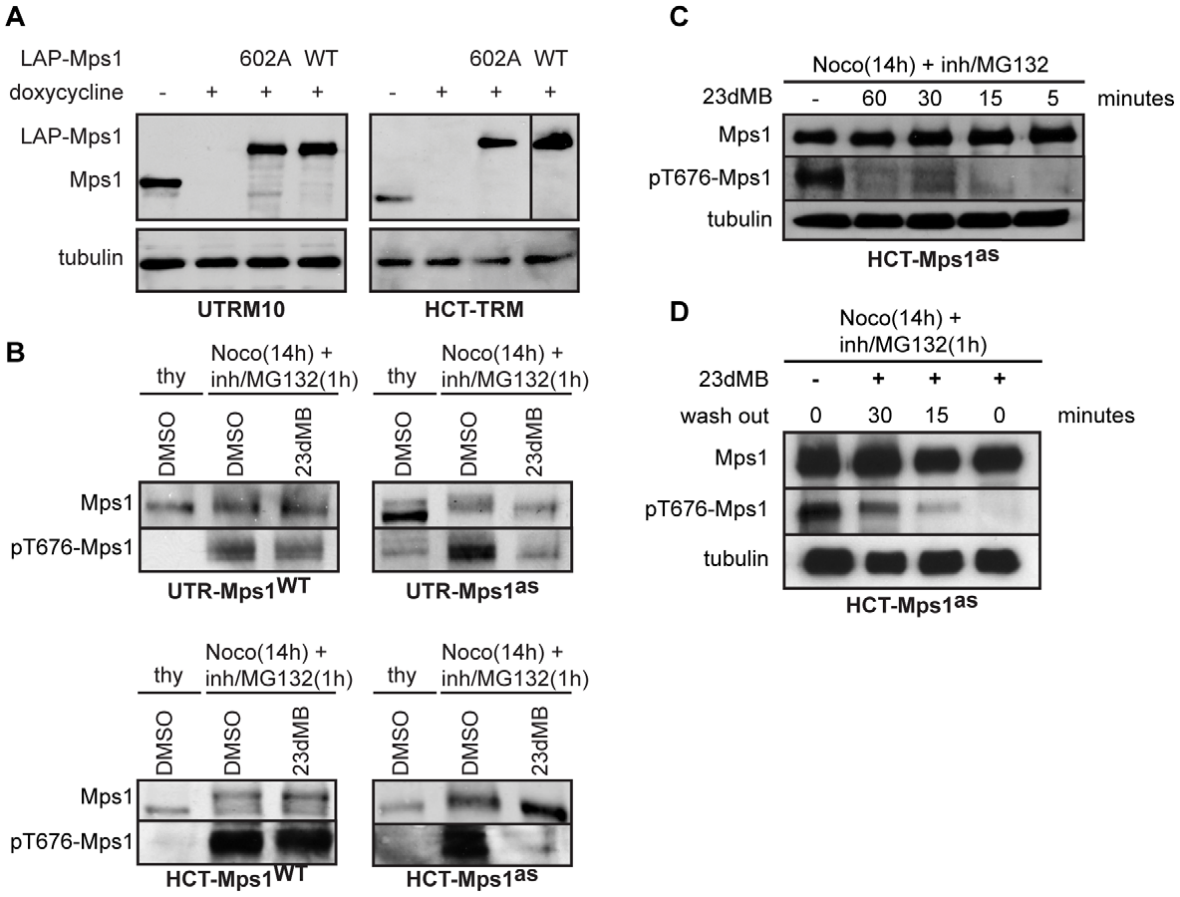
Stable expression of LAP-Mps1^as^ renders selective sensitivity to 23dMB-PP1. A: Mps1 and tubulin immunoblots of lysates of UTRM10 and HCT-TRM cells in which endogenous Mps1 is removed by doxycycline-induced expression of Mps1 shRNA and replaced with the indicated LAP-Mps1 mutants. B–D: pT676-Mps1 and Mps1 immunoblots of UTR-Mps1^as^ (B) and HCT-Mps1^as^ (B–D) cell lines treated with nocodazole and MG132 in combination DMSO/23dMB-PP1 for 1 hour (B), with nocodazole and MG132 for 1 hour in combination with an additional 23dMB-PP1 treatment for the indicated amounts of time (C), or with nocodazole and MG132 in combination with 23dMB-PP1 for 1 hour, followed by PBS wash after which cells were left for 15 or 30 minutes in media without 23dMB-PP1 (D).

To create stable cell systems in which Mps1 could be inhibited in a potent and reproducible manner, we stably expressed LAP-Mps1^M602A^ (hereafter referred to as Mps1^as^) or LAP-Mps1^M602G^ in UTRM10 (U2OS-derived) and HCT-TRM (HCT116-derived) cell lines in which endogenous Mps1 could be removed by doxycycline-induced expression of Mps1 shRNA [17]. Continuous growth in doxycycline and clonal selection resulted in stable, viable clonal cell lines in which endogenous Mps1 was undetectable and replaced with Mps1^WT^ or Mps1^as^ (Fig. 1A). No HCT-TRM clones expressing Mps1^M602G^ were obtained and only one such UTRM10 clone grew out. We suspect that the low activity of Mps1^M602G^ (see below) significantly decreased the chance of survival of cells expressing only this form of Mps1. Nevertheless, one UTRM10 cell line expressing Mps1^M602G^ was established, showing that under certain circumstances, Mps1^M602G^ can support viability. All cell clones (renamed HCT-Mps1^WT^, HCT-Mps1^as^, UTR-Mps1^WT^, UTR-Mps1^as^ and UTR-Mps1^M602G^) have been kept in culture for months, showing that replacement of Mps1 is stable.

Human Mps1 auto-activates by phosphorylating T676 in its activation loop. T676 phosphorylation is essential for full kinase activity [16], [22], [23], and the phosphorylation status of T676 serves as a read-out for Mps1 kinase activity [16]. To investigate if Mps1^as^ activity could be inhibited by bulky PP1 analogs in our engineered cell lines, we examined Mps1 T676 phosphorylation in different conditions. Following tests on the potency of different PP1 analogs to inhibit Mps1^as^ (Fig. S1B), we settled on 23dMB-PP1 (1-(*tert*-butyl)-3-(2,3-dimethylbenzyl)-1*H*-pyrazolo[3,4-*d*]pyrimidin-4-amine) as the Mps1^as^ inhibitor of choice. As expected, Mps1^WT^ and Mps1^as^ displayed a mobility shift and phosphorylation of T676 upon nocodazole addition in both HCT and UTR cell types (Fig. 1B). Whereas addition of 23dMB-PP1 did not affect the mobility or T676 phosphorylation of Mps1^WT^, 23dMB-PP1 prevented hyperphosphorylation as well as T676 phosphorylation of Mps1^as^ (Fig. 1B). Full inhibition of Mps1^as^ was established within 5 minutes after addition of 23dMB-PP1 (Fig. 1C). Furthermore, T676 phosphorylation could be detected 15 minutes after removal of 23dMB-PP1, showing that this rapid inhibition was reversible (Fig. 1D). Interestingly, very little T676 phosphorylation and weak mobility shift was detected in the UTR-Mps1^M602G^ clone, indicating this engineered Mps1 kinase has strongly reduced activity compared to Mps1^WT^ and Mps1^as^ (Fig. S2A). Although any residual activity and all Mps1 functions could be efficiently inhibited by treatment of the UTR-Mps1^M602G^ cells with PP1 inhibitors (Fig. S2 and S3B), we focused our investigations on studies with the Mps1^as^ clones.

### Mps1^as^ inhibition by 23dMB-PP1 disables mitotic checkpoint signaling

**Figure 2:**
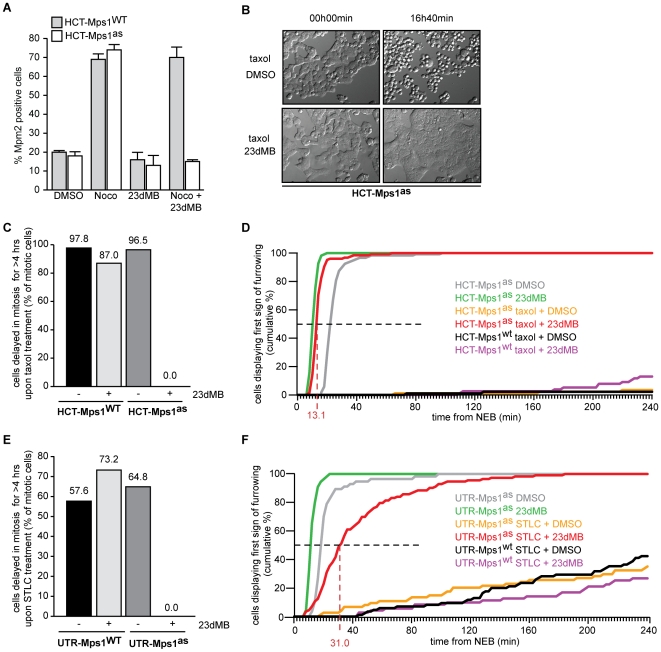
Mps1 activity is essential for the mitotic checkpoint. A: Quantification of flow cytometric analysis of the fraction of mitotic cells treated with DMSO or 23dMB-PP1 alone or in combination with nocodazole for 14 hours. Graph represents average of three experiments (+/−SD). B–F: Time-lapse analysis by DIC microscopy of HCT- and UTR-derived cells treated with taxol (HCT-derived cells) or STLC (UTR-derived cells) in combination with DMSO or 23dMB-PP1. Images in B display morphology of HCT-Mps1^as^ cells imaged at the indicated timepoints after addition of taxol in combination with DMSO or 23dMB-PP1. Graphs in C and E represent percentages of cells delayed in mitosis for 4 hours or longer. Percentages are indicated at top of each column. Line graphs in D and F show cumulative percentages of cells that display first signs of furrowing at indicated times after NEB. Dashed lines indicate half-time to furrowing of inhibited drug-treated Mps1^as^ cells.

Mps1 kinase activity is needed for the mitotic checkpoint to delay mitosis when unattached kinetochores persist [6], [9], [12], [18], [21]. We next studied the ability of the cell lines to maintain a mitotic checkpoint response in the presence of 23dMB-PP1. In absence of 23dMB-PP1, all HCT-Mps1^WT^ and HCT-Mps1^as^ cell lines sustained checkpoint activity in the presence of nocodazole as scored by mitotic index (Fig. 2A). The ability to maintain a nocodazole-induced mitotic delay was not lost in Mps1^WT^ cells upon treatment with 23dMB-PP1, showing 23dMB-PP1 did not have off-target effects that could disable the mitotic checkpoint. In contrast to Mps1^WT^-expressing cells, 23dMB-PP1 completely abolished mitotic checkpoint activity in Mps1^as^ cells. Similar results were obtained with the UTR-Mps1^M602G^ cells (Fig. S2B).

To ensure that Mps1 inhibition decreased mitotic index by inhibiting mitotic checkpoint activity rather than, for instance, preventing entry into mitosis, mitotic progression in the presence of spindle poisons was monitored by live cell differential interference contrast (DIC) microscopy. As a measure for time spent in mitosis, the time from nuclear envelope breakdown (NEB) to the first signs of furrow ingression was scored. Engaging the mitotic checkpoint in these cells by treatment with the spindle drugs nocodazole or taxol, or the Eg5 inhibitor STLC caused all cell lines to delay mitosis for hours (Fig. 2B–F and S3). Delays were most pronounced in cells with the HCT-TRM background where ∼95% of cells maintained the arrest for at least 4 hours (Fig. 2C, D and S3A, C) and ∼55% for at least 10 hours (data not shown). In stark contrast, addition of 23dMB-PP1 caused all HCT-Mps1^as^ but not HCT-Mps1^WT^ cells to exit mitosis within 60 minutes (Fig. 2D and S3A, C). These data show that the ability of cells to delay mitotic progression absolutely requires Mps1 kinase activity. Moreover, the finding that all 23dMB-PP1-treated HCT-Mps1^as^ cells had lost this ability illustrated the clonality of this cell line.

### Mps1 kinase activity promotes localization of Mad1, Mad2, Cdc20 and Bub1 to unattached kinetochores

Proper functioning of the mitotic checkpoint requires the recruitment of checkpoint proteins to the kinetochore. Kinase activity of Mps1 is required for the recruitment of Mad2 to unattached kinetochores, at least partly explaining its necessity for checkpoint signaling [9], [12], [21]. There is, however, discrepancy between reports on the requirement of Mps1 for the recruitment of Mad1 [11], [12], [21], [24], Bub1 [11], [12], [24], [25], BubR1 [11], [12], [18], [23] and CENP-E [9], [12], [21], [24], [26] to kinetochores.

**Figure 3:**
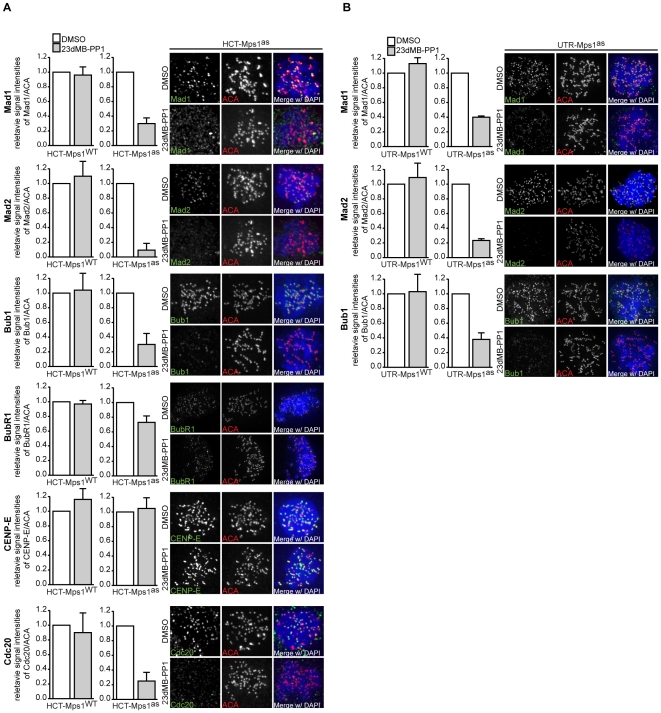
Mps1 kinase activity promotes localization of Mad1, Mad2, Bub1 and Cdc20 to unattached kinetochores. A, B: Immunolocalization of Mad1, Mad2, Bub1, BubR1, CENP-E, Cdc20, and centromeres (ACA) in HCT-derived (A) or UTR-derived (B) cells treated with nocodazole and MG132 for 1 hour in combination with DMSO or 23dMB-PP1. Graphs represent quantifications of kinetochore signal intensities for the investigated proteins at all kinetochores in a single cell as a ratio of the ACA signal. Data are average of three experiments, 10 cells per experiment (+/− SD). Representative images are shown.

To examine how Mps1 catalytic activity contributes to recruitment of these and other checkpoint proteins, we assayed their kinetochore association after inhibition of Mps1^as^ in our cell lines. Like the ability to delay mitosis, kinetochore recruitment of Mad1, Mad2, BubR1, Bub1, CENP-E and Cdc20 was altered neither in DMSO-treated Mps1^WT^ or Mps1^as^ cells, nor in 23dMB-PP1-treated Mps1^−WT^ cells (Fig. 3 and S4). However, addition of 23dMB-PP1 to Mps1^as^ cells significantly reduced the level of Mad1, Mad2, Bub1 and Cdc20 at unattached kinetochores, but only marginally affected BubR1 levels and did not influence CENP-E levels (Fig. 3). These data show that Mps1 kinase activity promotes kinetochore-binding of Mad1, Mad2, Bub1 and Cdc20.

### Mps1 inhibition strongly diminishes MCC levels in mitotic cells

**Figure 4:**
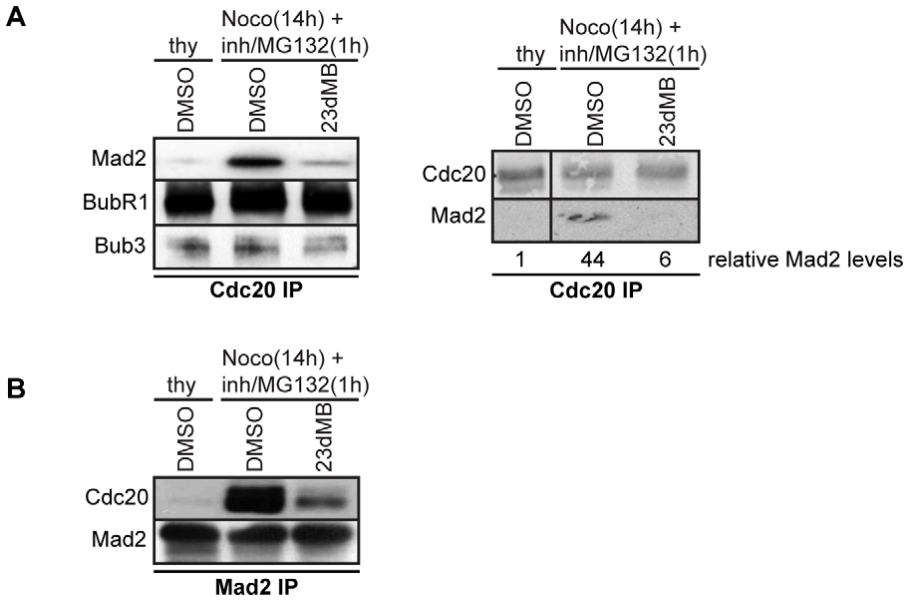
Mps1 inhibition reduces Mad2-Cdc20 interaction. A, B: Immunoblots of the indicated proteins in immunoprecipitations of Cdc20 (A) or Mad2 (B). Immunoprecipitations were preformed on interphase cell lysates (thymidine, thy) or mitotic cell lysates (nocodazole, noco) of HCT-Mps1^as^ cells treated with DMSO or 23dMB-PP1 in combination with MG132 for 1 hour. Quantification of Mad2 levels in Cdc20 immunoprecipitates, relative to interphase levels, is indicated in A, right panel.

The mitotic checkpoint functions through the production of an inhibitory complex called the mitotic checkpoint complex (MCC). The MCC targets Cdc20, the main activator of the anaphase promoting complex or cyclosome (APC/C) in prometaphase, thereby preventing exit from mitosis [2]. The amplification of MCC assembly by unattached kinetochores is dependent on kinetochore localization of Mad1 and Mad2 [27], [28]. Since inhibition of Mps1 resulted in loss of kinetochore-bound Mad1/Mad2, we set out to investigate if Mps1 inhibition influences the amount of MCC in nocodazole-treated cells. As expected, Cdc20 immunoprecipitations from cells treated with DMSO contained the MCC subunits Mad2, BubR1 and Bub3 (Fig. 4A). Although inhibition of Mps1 did not alter the levels of coprecipitated BubR1 and Bub3, the amount of Cdc20-bound Mad2 in mitotic cells was reduced to interphase levels after treatment with 23dMB-PP1 (Fig. 4A). Similarly, Mad2 immunoprecipitations displayed little bound Cdc20 in cells treated with 23dMB-PP1 when compared to DMSO-treated cells (Fig. 4B). These data show that Mps1 catalytic activity is needed for formation and/or stability of MCC.

### Mps1 inhibition decreases the time spent in mitosis

**Figure 5:**
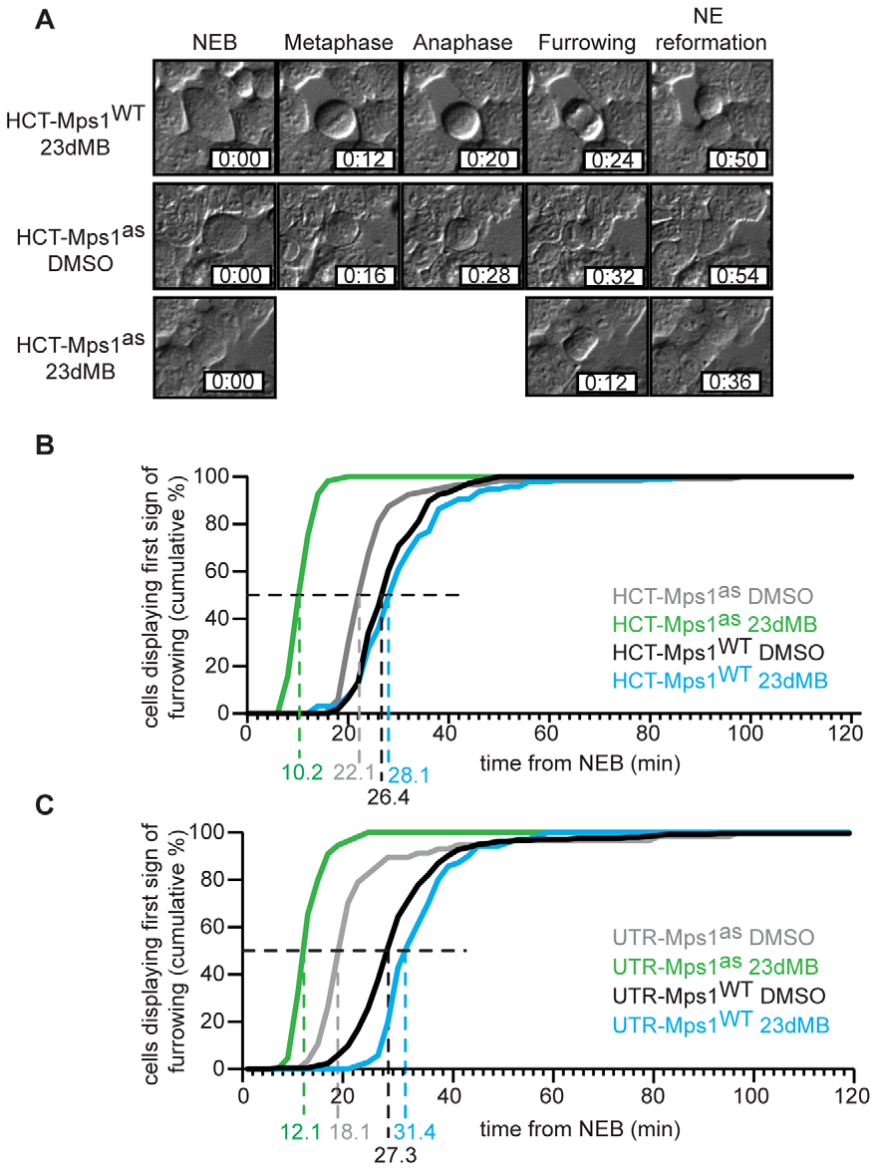
Mps1 inhibition accelerates mitosis. A–C: Time-lapse analysis by DIC microscopy of mitotic progression of HCT-derived (A, B) and UTR-derived (C) cells treated with DMSO or 23dMB-PP1. Images in A display morphology of HCT-derived cells imaged at the indicated timepoints after NEB (nuclear envelope breakdown). Graphs in B and C show cumulative percentages of cells that display first signs of furrowing at indicated times after NEB. Dashed lines indicate half-time to furrowing.

To get more detailed insight in the functions of Mps1 kinase activity during mitosis, we examined various aspects of chromosome segregation in our cell lines by time-lapse microscopy. Mps1^WT^ cells treated with DMSO or 23dMB-PP1 progressed through mitosis normally. In addition, 23dMB-PP1 treatment of Mps1^as^-expressing cells caused no overt problems in mitotic entry, bipolar spindle formation or cytokinesis in neither cell line. Strikingly, however, 23dMB-PP1-treated Mps1^as^ cells sped through mitosis (Fig. 5A). To more carefully quantify this phenotype, the time from NEB to the first signs of furrowing was measured. Half of DMSO-treated HCT-Mps1^WT^ and HCT-Mps1^as^ cells had proceeded to furrow ingression 26 and 22 minutes after NEB, respectively (Fig. 5B). Strikingly, whereas addition of 23dMB-PP1 had no effect on the time to furrow ingression of HCT-Mps1^WT^ cells, half the 23dMB-PP1-treated HCT-Mps1^as^ cells showed signs of furrow ingression in as little as 10 minutes and no cell took longer than 20 minutes to reach this furrowing stage of mitosis (Fig. 5A, B). Further evidence for the high penetrance of Mps1 inhibition in these cells was illustrated by the fact that exit from mitosis in taxol was within a similar timeframe as these unperturbed mitosis: 13 vs. 10 minutes, respectively (Fig. 2D). A similar, albeit less consistent effect was apparent in the UTR-derived cell lines: Half-time to furrowing in UTR-Mps1^as^ cells was reduced from 18 to 12 minutes by treatment with 23dMB-PP1, whereas this was not altered by 23dMB-PP1 in UTR-Mps1^WT^ cells (Fig. 5C).

### Mps1 activity facilitates attachment error-correction

**Figure 6:**
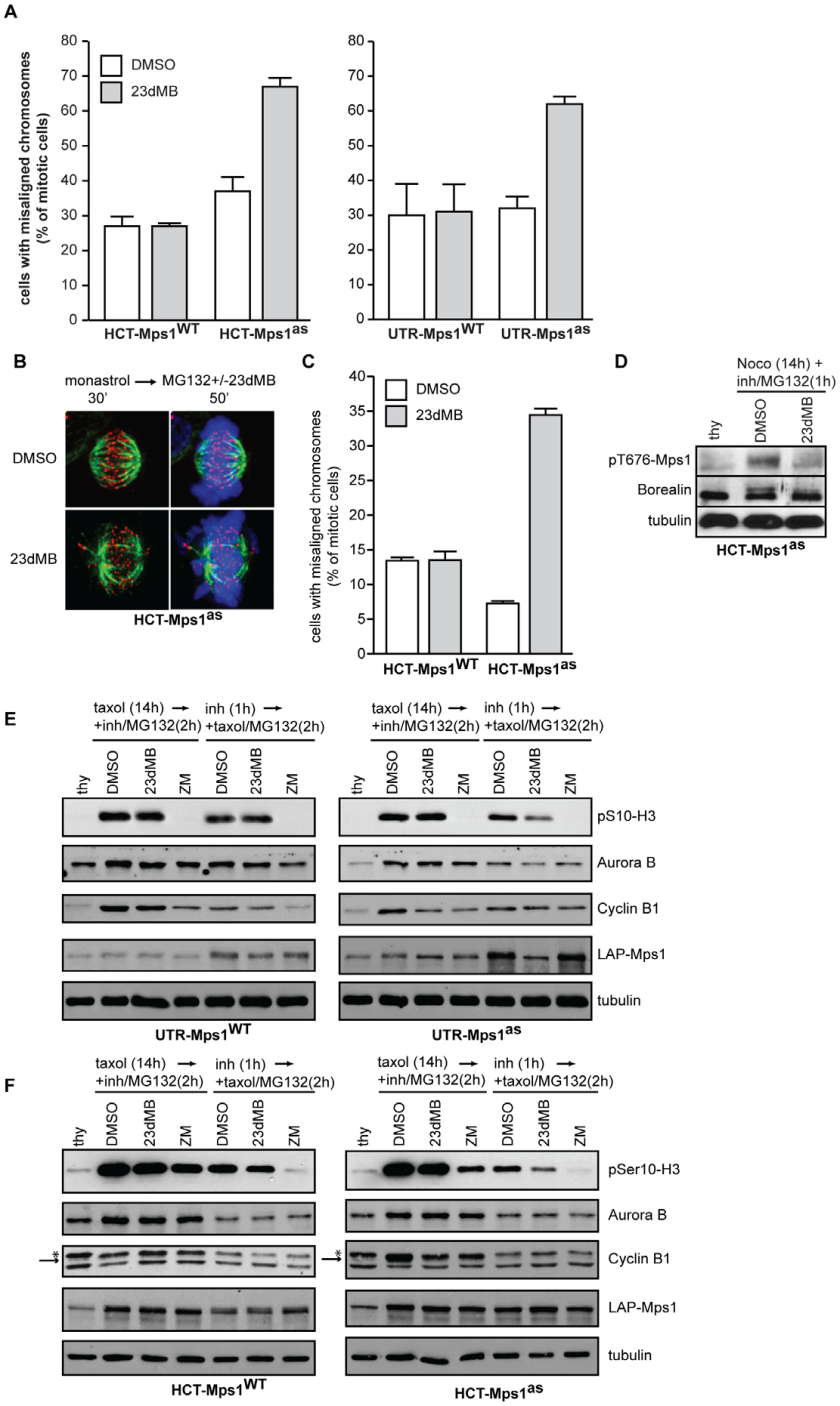
Mps1 activity is required for efficient correction of erroneous attachments. A: Chromosome alignment in HCT- and UTR-Mps1^WT^ and -Mps1^as^ cell lines treated with MG132 for 60 minutes in combination with DMSO or 23dMB-PP1. Graphs display percentages of cells with misaligned chromosomes, averages of three experiments, at least 50 mitotic cells per experiment (+/−SD). B, C: Immunostainings of HCT-Mps1^WT^ and HCT-Mps1^as^ cells treated with monastrol for 30 min followed by a release from monastrol into MG132 with or without 23dMB-PP1 for 50 minutes. Cells in B were immunostained for tubulin (green) and centromeres (ACA, red). DNA (DAPI) is in blue. Graph in C indicates the percentage of mitotic cells with misaligned chromosomes 50 minutes after monastrol washout, averages of two experiments, at least 100 mitotic cells per experiment (+/−SD). D: Immunoblots of Borealin after phosphate-affinity gel electrophoresis of lysates from nocodazole-treated HCT-Mps1^as^ cells to which DMSO or 23dMB-PP1 and MG132 was added during the final hour. E, F: Immunoblots of the indicated proteins in lysates of the UTR- (E) and HCT-Mps1 (F) variant cell lines. Cells were treated either with thymidine (thy), with taxol for 14 hours followed by the indicated inhibitors for an additional 2hrs, or with the indicated inhibitors for 1 hour followed by taxol/MG132 treatment for an additional 2 hrs ZM, ZM447439. Arrow in F points to Cyclin B. Asteriks indicates aspecific band in thymidine sample.

Mps1 is critical for chromosome biorientation in yeast and humans [12], [13] and our previous results using Mps1 RNAi supported the hypothesis that Mps1 facilitates error-correction by promoting Aurora B activity at inner centromeres [12]. Nevertheless, a true display of the role for Mps1 in error-correction requires inhibition of Mps1 activity solely during the error-correction process. Our analog-sensitive cell lines provided the opportunity to test this. We first verified that Mps1 inhibition prevented efficient chromosome alignment on the cell's equator. Cells were treated with MG132 for 60 minutes to prevent exit from mitosis and provide cells with sufficient time to align all chromosomes. Roughly 30% of DMSO- or 23dMB-PP1-treated HCT-Mps1^WT^ cells or DMSO-treated HCT-Mps1^as^ cells displayed misaligned chromosomes within this time (Fig. 6A). In agreement with our previous studies [12], addition of 23dMB-PP1 to HCT-Mps1^as^ cells caused severe alignment problems in ∼70% of cells. Similar results were obtained by Mps1 inhibition in UTR-Mps1^M602G^ cells (Fig S2C).

We next investigated if the generated cells lines were able to correct faulty attachments when Mps1 was inhibited during error-correction rather than prior to the establishment of errors. To this end, cells were treated with the Eg5-inhibitor monastrol to create monopolar spindles with large numbers of chromosomes that have syntelic or monotelic attachments [29]. Monastrol can efficiently be removed from cell, which allows the formation of a bipolar spindle in which full chromosome alignment requires correction of the improper attachments by Aurora B [30], [31]. All attachment errors were corrected and full chromosome alignment was restored in 87% of HCT-Mps1^WT^ cells 50 minutes after removal of monastrol (Fig. 6B, C). Addition of 23dMB-PP1 during monastrol washout of Mps1^as^ cells however, caused significant problems with chromosome alignment (Fig. 6B, C). Importantly, the non-aligned chromosomes were often seen to have retained syntelic attachments, indicative of error-correction deficiency. These data solidify the notion that Mps1 activity is needed for efficient correction of erroneous attachments.

### Mps1 enhances Aurora B activity

Erroneous kinetochore-microtubule attachments cause a lack of tension between sister centromeres which allows Aurora B kinase activity to destabilize such attachments [31]–[36]. We and others previously found that Mps1 enhances Aurora B activity up to 4-fold by phosphorylating the CPC component Borealin, explaining the contribution of Mps1 to error-correction [12], [14]. We next set out to examine the link between Mps1 and Aurora B activity in our analog-sensitive cell lines. Using phosphate-affinity gel electrophoresis [37], Borealin displayed a prominent mobility shift in mitotic samples of nocodazole-treated cells (Fig. 6D). This shift was reduced upon inhibition of Mps1^as^ by 23DMB-PP1, showing for the first time that phosphorylation of endogenous Borealin is under control of Mps1 activity.

To investigate the consequences of Mps1 inhibition on Aurora B activity, mitotic cells were collected by shake-off after 14 hours of taxol treatment and subsequently treated with 23dMB-PP1 or DMSO in combination with MG132 for an additional hour, after which mitotic lysates were examined for phosphorylation of Serine 10 in Histone H3 (pS10-H3) by immunoblot. Surprisingly, addition of 23dMB-PP1 to UTR- or HCT-Mps1^as^ cells that had been arrested in mitosis for hours did not significantly reduce pS10-H3, even though Mps1 activity, as measured by mobility shift, was inhibited (Fig 6E, F). These findings are in seeming contrast to our previous report, in which Mps1 RNAi reduced pS10-H3 levels in HeLa cells [12]. Since one difference between these experiments is whether or not cells entered mitosis with intact Mps1 activity, we next examined pS10-H3 levels in cells that had seen 23dMB-PP1 prior to mitotic entry. For this, cells were treated with DMSO or 23dMB-PP1 for 1 hour, after which taxol/MG132 was added for an additional 2 hours. Analysis of these mitotic samples showed significant reduction of pS10-H3 levels in 23dMB-PP1-treated Mps1^as^ cells but not Mps1^WT^ cells (Fig. 6E, F and S5). The magnitude of this decrease is less prominent than direct inhibition of Aurora B activity using ZM447439 (Fig. 6E, F and S5) [33], which is in line with our previous observations that showed that while Mps1 could enhance Aurora B activity, it was not absolutely critical [12].

### Selective, short-term inhibition of Mps1 kills cells

**Figure 7:**
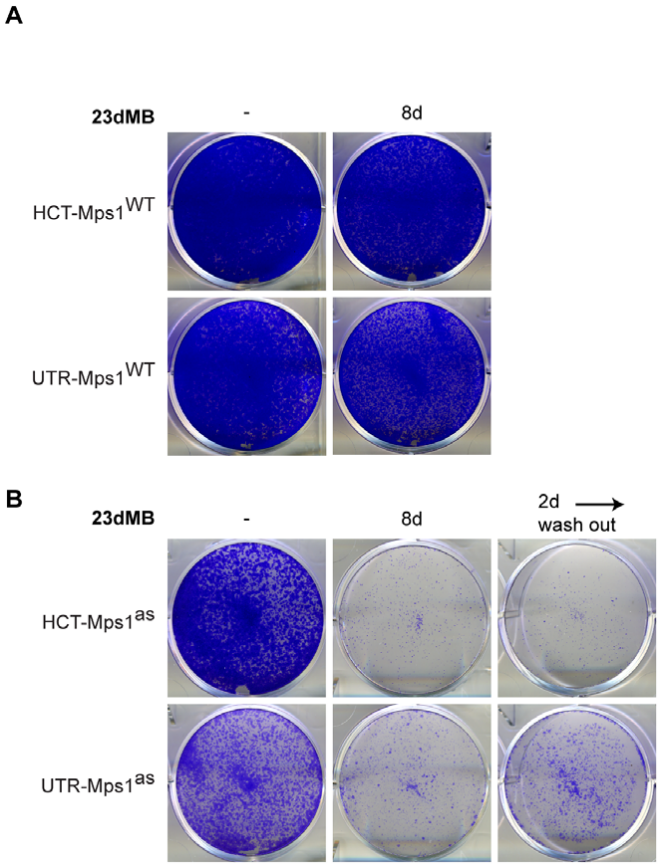
Specific inhibition of Mps1 enzymatic activity causes cell death. HCT-Mps1^WT^ and UTR-Mps1^WT^ cells (A) and HCT-Mps1^as^ and UTR-Mps1^as^ cell (B) were treated with or without (-) 23dMB-PP1 for 8 days (8d), or with 23dMB-PP1 for 2 days (2d) followed by wash-out and continued growth until day 8.

Long-term Mps1 RNAi kills U2OS, HCT116 and LS174T cells [16], [17] and Mps1 inhibition has been proposed as an effective way to induce a lethal dose of chromosome segregation errors [17], [38], [39]. To examine if selective and penetrant inhibition of the catalytic activity of Mps1 is lethal, UTR- and HCT-derived Mps1^wt^ and Mps1^as^ cells were treated with 23dMB-PP1 for 8 days after which colony outgrowth was scored. 23dMB-PP1 had no off-target effect on viability during this period, as Mps1^wt^ cells remained fully viable despite prolonged exposure to 23dMB-PP1 (Fig. 7A). As shown in Fig. 7B, however, no Mps1^as^ cells survived prolonged Mps1 inhibition. To approach a more relevant duration of inhibition in potential future clinical settings, we next tested the effect of short-term inhibition of Mps1 on cell viability. Addition of 23dMB-PP1 for 2 days and subsequent removal of the inhibitor until day 8 left no (HCT-Mps1^as^) or very few (UTR-Mps1^as^) cells alive (Fig. 7B). These data show that efficient inhibition of Mps1 for a relatively short period of time is sufficient to kill cells.

## Discussion

In the present study we investigated the different roles of Mps1 in mitosis of human cells through the use of chemical genetics. We report here two clonal cell lines expressing analog-sensitive versions of Mps1 in which endogenous Mps1 was stably removed by doxycycline-induced shRNA expression. Use of these cell lines circumvents experimental limitation in reproductivity and penetrance associated with transient RNAi reconstitutions [21] and allows temporal control of Mps1 activity.

Chemical genetic inhibition of Mps1 was previously used to uncover novel aspects of Mps1 in *S. cerevisiae* mitosis [6], [13]. A yeast strain carrying the analog-sensitive M516G mutation (similar to the M602G mutation in human Mps1, included in the present study) allowed analysis of Mps1 functions after the moment of spindle pole body duplication, a critical function of Mps1 in budding yeast [4], [6]. Using this strain (*mps1-as1*), it was verified that Mps1 inhibition prevents mitotic checkpoint activity and causes cell death [6]. In addition, Mps1-inhibited yeast cells were found to display spindle morphology defects and chromosome mispositioning [6], possibly as a consequence of errors in chromosome biorientation [13].

In agreement with these and other reports [6], [12], [13], chemical genetic inhibition of Mps1 in our human cell lines verified that Mps1 kinase activity is critical for chromosome biorientation. Although our data using Mps1 RNAi supported the hypothesis that Mps1 facilitated error-correction by promoting Aurora B activity at inner centromeres [12], we now show that error-correction is also prevented when Mps1 kinase activity is inhibited only during the error-correction process in cells undergoing spindle bipolarisation after monastrol wash-out. Additionally, through the use of phosphate-affinity gel electrophoresis we show that phosphorylation of endogenous Borealin is under the control of Mps1. Borealin phosphorylation enhances Aurora B activity [12], [14] yet inhibition of Mps1 did not affect pS10-H3 levels under conditions that showed an increase in Borealin mobility. pS10-H3 was only found reduced when cells entered mitosis without Mps1 activity. These results suggest that partial reduction in Aurora B activity caused by Mps1 inhibition is difficult to detect once pS10-H3 is fully established, possibly because of low turnover of the phosphate group on S10-H3. In support of this, inactivation of Aurora B by a 2 hour exposure of our HCT-derived cells to ZM447439 only caused a moderate reduction of pS10-H3 levels. These data indicate that pS10-H3 serves as a good readout for Mps1-dependent regulation of Aurora B activity when pS10-H3 is not fully established at mitotic entry. Nevertheless, since Mps1 inhibition during mitosis affects Borealin phosphorylation and error-correction, it is likely that acute effects on Aurora B activity by Mps1 inhibition can be visualised with antibodies to the relevant error-correction substrates.

The function of Mps1 kinase activity in the regulation of the mitotic checkpoint has previously been established by various labs including ours, and the present data fully agree with this [6], [9], [12], [18], [21]. As expected [11], [12], [24], Mps1 inhibition in our cell lines caused mislocalization of Mad1 and Mad2. This disagrees with the study by Tighe et al., in which chemical genetic inhibition of Mps1 in cells transiently transfected with Mps1 shRNA plasmids had little effect on Mad1 recruitment [21]. This discrepancy may be due to differences in penetrance of Mps1 inhibition. Whereas inhibition of Mps1 with 1NM-PP1 in the Tighe et al. study left mitotic progression unaffected in a large proportion of cells, mitosis was disturbed in the vast majority (UTR-Mps1^as^) or all (HCT-Mps1^as^) cells in our cell lines (Fig. 2 and 5). This difference in penetrance may be due to inefficient RNAi by transient shRNA transfection or potency of 1NM-PP1 in the Tighe et al., study. In our hands, 1NM-PP1 was significantly less capable of inhibiting Mps1 than 23dMB-PP1 or 3MB-PP1 (Fig. S2). Besides Mad1/Mad2, our data further indicate that Mps1 is essential for recruitment of Cdc20 as well as Bub1 to unattached kinetochores, in agreement with some [11], [25] but not other [12], [24] previous reports using Mps1 RNAi or Mps1 protein depletion.

Mps1 inactivation affects the quality of chromosome segregation in unperturbed mitosis [16], [21]. Using time-lapse microscopy we show that inactivation of Mps1 accelerates mitosis. A similar phenotype was reported for cells depleted of Mad2 or BubR1 [40]. The regulation of mitotic timing by Mad2 and BubR1 is kinetochore-independent, since Mad1 depletion, while disabling the mitotic checkpoint, did not affect normal mitotic timing [40]. It is therefore unlikely that lack of recruitment of Mad1 and Mad2 to kinetochores in Mps1-inhibited cells contributes to accelerated mitosis. An explanation for the timing phenotype, proposed by Meraldi et al., and in agreement with experimental data [41], is the possibility that sufficient MCC exists in interphase to constrain APC/C activity long enough to allow full chromosome biorientation in the majority of cells. In this model, only presence of persistent unattached kinetochores requires additional MCC formation to further prolong mitosis. Given the timing phenotype of Mps1-inhibited cells, it is therefore possible that Mps1, in addition to influencing Mad1-dependent MCC formation, affects stability or inhibitory potential of pre-assembled MCC. Further studies are needed to examine this hypothesis.

Due to its central role in mitosis, Mps1 is an attractive target for anti-cancer therapies. A recent study from our lab proposed that elevating the frequency of chromosome segregation errors by partial Mps1 reduction can be of clinical relevance, especially with other treatments that affect fidelity of chromosome segregation [17]. We show here for the first time that specific inhibition of Mps1 enzymatic activity efficiently kills human cells. Moreover, a short pulse of inhibition followed by recovery caused lethality in a significant fraction of cells. As such a pulse of Mps1 inhibition is more relevant to potential future clinical situations than potent, long-term kinase inhibition, these data illustrate that specific and penetrant inhibition of Mps1 activity might indeed be a promising anti-cancer strategy.

## Materials and Methods

### Tissue Culture and Treatments

UTR-Mps1^WT^, UTR-Mps1^as^, HCT-Mps1^WT^ and HCT-Mps1^as^ cells were derived from UTRM10 [11], [16], [17] and HCT-TRM (named HCT-116-TetRMps1 clone#2 in Ref. [17]) cell lines, respectively. Briefly, these cell lines stably express TetR and carry pSuperior-retro-puro-Mps1, resulting in depletion of Mps1 protein upon addition of doxycycline. To generate the derivative cell lines that express Mps1^WT^ and Mps1^as^, UTRM10 and HCT-TRM cells were transfected using the calcium phosphate method with the different LAP-Mps1 alleles and selected with doxycycline (1 µg/ml). Single colonies were selected after limiting dilution. HCT-TRM-derivative cells were grown in RPMI (Lonza) with 6% Tet-approved FCS (Clontech), supplemented with pen/strep (Invitrogen). UTRM10-derivative cells were grown in DMEM (Lonza) with 8% Tet-approved FCS (Clontech), supplemented with pen/strep (Invitrogen) and Ultra-Glutamine (Lonza).

Thymidine (2.5 mM), nocodazole (660 nM), taxol (1 µM), MG132 (10 µM), monastrol (200 µM), STLC (10 µM), doxycycline and puromycin (1 µg•ml^−1^) were all from Sigma, ZM447449 (Tocris Bioscience) was added at 2 µM and 23dMB-PP1 at 1 µM.

### Antibodies

The following primary antibodies were used for immunoblotting, immunofluorescence imaging and FACS analyses: Mps1-NT (Upstate), pT676-Mps1 [16], BubR1-300A (Bethyl), Mad2 (custom polyclonal Rb antibody. Briefly, His_6_-Mad2 (pQE80L-hMad2) was expressed in BL21 cells, purified using Ni-agarose (Qiagen) and injected into New Zealand rabbits (Covance). Mad2 antibody was affinity purified from rabbit serum using His_6_-Mad2), Bub1 (Abcam), Mad1 (SantaCruz Biotechnology), α-tubulin (Sigma), CREST (Cortex Biochem), MPM2 (Upstate), Cdc20 (SantaCruz Biotechnology), Bub3 (BD Trans Lab) and Borealin (a gift of W. Earnshaw). Secondary antibodies included anti-human Alexafluor647 and anti-rabbit Alexafluor488 (Molecular Probes) for immunofluorescence studies, anti-mouse-Cy5 (Jackson) for FACS and anti-Mouse/Rabbit Alexa680/800 (Molecular Probes) for immunoblotting.

### Flow cytometry

HCT- and UTR-derived cell lines were released from a 24 hours thymidine block into nocodazole or 23dMB-PP1 or a combination of both for 18 hours, harvested and fixed in 70% ice-cold ethanol for 24 hours, and analyzed using MPM2 immunostains as described [42].

### Immunofluorescence and live cell imaging

For immunolocalization studies, cells were plated on 12 mm coverslips, pre-extracted with 0.2% TritonX-100 in warm PEM (100 mM PIPES pH6.8, 1mM MgCl2 and 5mM EGTA) for 1 minute, fixed with 3% PFA in PBS, blocked with 3% BSA in PBS for 1 hour, incubated with primary antibody for 16 hrs at 4°C, washed with PBS/0.1% TritonX-100 and incubated with secondary antibodies for an additional 1 hour at room temperature. Coverslips were washed and submerged in PBS containing DAPI, washed again and mounted using ProLong Antifade (Molecular Probes). All images were acquired on a DeltaVision RT system (Applied Precision) with a 100X/1.40NA UPlanSApo objective (Olympus) using SoftWorx software. Images are maximum intensity projections of deconvolved stacks. For quantification, all images of similarly stained experiments and acquired with identical illumination-settings were analyzed using ImageJ. Average pixel intensities of the total of all regions encompassing centromeres plus kinetochores were determined in the various channels and corrected for background.

For live cell imaging, cells were plated in 4-well chambered glass-bottom slides (LabTekII) or 24-well glass bottom plates (MatTek), transfected and imaged in a heated chamber (37°C and 5% CO_2_) using a 20X/0.5NA UPLFLN objective on a Olympus IX-81 microscope, controlled by Cell-M software (Olympus). 16 bits DIC (5 msec exposure) images were acquired every 2 minutes using a Hamamatsu ORCA-ER camera. Images were processed using Cell-M software.

### Immunoprecipitation

HCT-Mps1^as^ cells were released from a 24 hours thymidine block into nocodazole for 18 hours, harvested and lysed in 50 mM Tris 7.5, 150 mM NaCl, 1% TX-100, 2 mM MgCl2, 5 mM EDTA, supplemented with protease inhibitors and phosphatase inhibitors for 10 minutes on ice. The cleared extract was incubated with 10% protein A-agarose beads (Roche)/antibody mix for 2 hours at 4°C on a rotating wheel. The beads were washed twice with lysis buffer. Supernatant and beads were processed for SDS-PAGE and the proteins were transferred to nitrocellulose membranes for immunoblotting.

### Colony Formation Assays

Cells (50,000/well) were plated in 6-well plates (Costar). Cells were grown in media supplemented with DMSO or 23dMB-PP1 (1 µM) for 8 days or for 2 days followed by inhibitor wash-out and continued incubation in media without 23dMB-PP1 for an additional 6 days. At day 8, plates were washed with PBS, fixed for 5 minutes with 96% methanol and stained with 0.1% crystal violet in water for 30 minutes.

## Supporting Information

Figure S1 A: Quantification of flow cytometric analysis of fraction of mitotic cells treated with nocodazole for 18 hours. Cells were transiently transfected with indicated LAP-Mps1 constructs. D664A is an enzymatically inactive mutant. B: Box-and-whisker diagrams of time spent in mitosis of UTR-Mps1as cell treated with taxol in combination with increasing concentrations of different bulky PP1 inhibitors. ND; not determined. (Molecular structures displayed in right panel.)
(0.27 MB TIF)

Figure S2 A: pT676-Mps1 and Mps1 immunoblot of UTR-Mps1WT, UTR-Mps1as and UTR-Mps1M602G cells treated with thymidine (thy) for 20 hours or with taxol and MG132 in combination with indicated inhibitors for 1 hour. 1NM, 1NM-PP1; 3MB, 3MB-PP1; SP, SP600125. B: Quantification of flow cytometric analysis of fraction of mitotic UTRM10, UTR-Mps1WT, UTR-Mps1as and UTR-Mps1M602G cells treated with taxol in combination with indicated inhibitors for 17 hours. C: Chromosome alignment in UTRM10, UTR-Mps1WT, UTR-Mps1as and UTR-Mps1M602G cells treated with MG132 for 50 minutes in combination with the indicated inhibitors. Graph displays percentages of cells with misaligned chromosomes.
(0.84 MB TIF)

Figure S3 A–C: Time-lapse analysis by DIC microscopy of HCT- and UTR-derived cells treated with (A) taxol (HCT-derived cells) or STLC (UTR-derived cells) in combination with DMSO or 23dMB-PP1, or nocodazole (B, C) in combination with DMSO or 23dMB-PP1. Graphs in A display the time from NEB to the first signs of furrow ingression for individual cells. Black bars indicate end of time-lapse acquisition. Line graphs in B and C show cumulative percentages of UTR-derived cells (B) and HCT-derived cells (C) displaying first signs of furrowing at indicated times after NEB.
(0.79 MB TIF)

Figure S4 Immunolocalization of Mad1, Mad2, Bub1, BubR1, Cdc20 and CENP-E and centromeres (ACA) in HCT-Mps1WT (A) and UTR-Mps1WT (B) cells treated with nocodazole and MG132 for 1 hour in combination with DMSO or 23dMB-PP1. Graphs represent quantifications of kinetochore signal intensities for the investigated proteins at all kinetochores in a single cell as a ratio of the ACA signal. Data are average of three experiments, 10 cells per experiment (±SD). Representative images are shown. DNA (DAPI) is in blue.
(3.76 MB TIF)

Figure S5 Immunoblots of the indicated proteins in lysates of the HCT- and UTR-Mps1 variant cell lines. Cells were treated with thymidine (thy), or with the indicated inhibitors for 1 hour followed by taxol/MG132 treatment for an additional 2 hrs. ZM, ZM447439.
(0.71 MB TIF)
