# Supplementary figures and images for: Chemical Genetic Inhibition of Mps1 in Stable Human Cell Lines Reveals Novel Aspects of Mps1 Function in Mitosis
Source: PLoS One. 2010 Apr 22;5(4):e10251. doi: 10.1371/journal.pone.0010251 (PMC2858645; doi:10.1371/journal.pone.0010251)

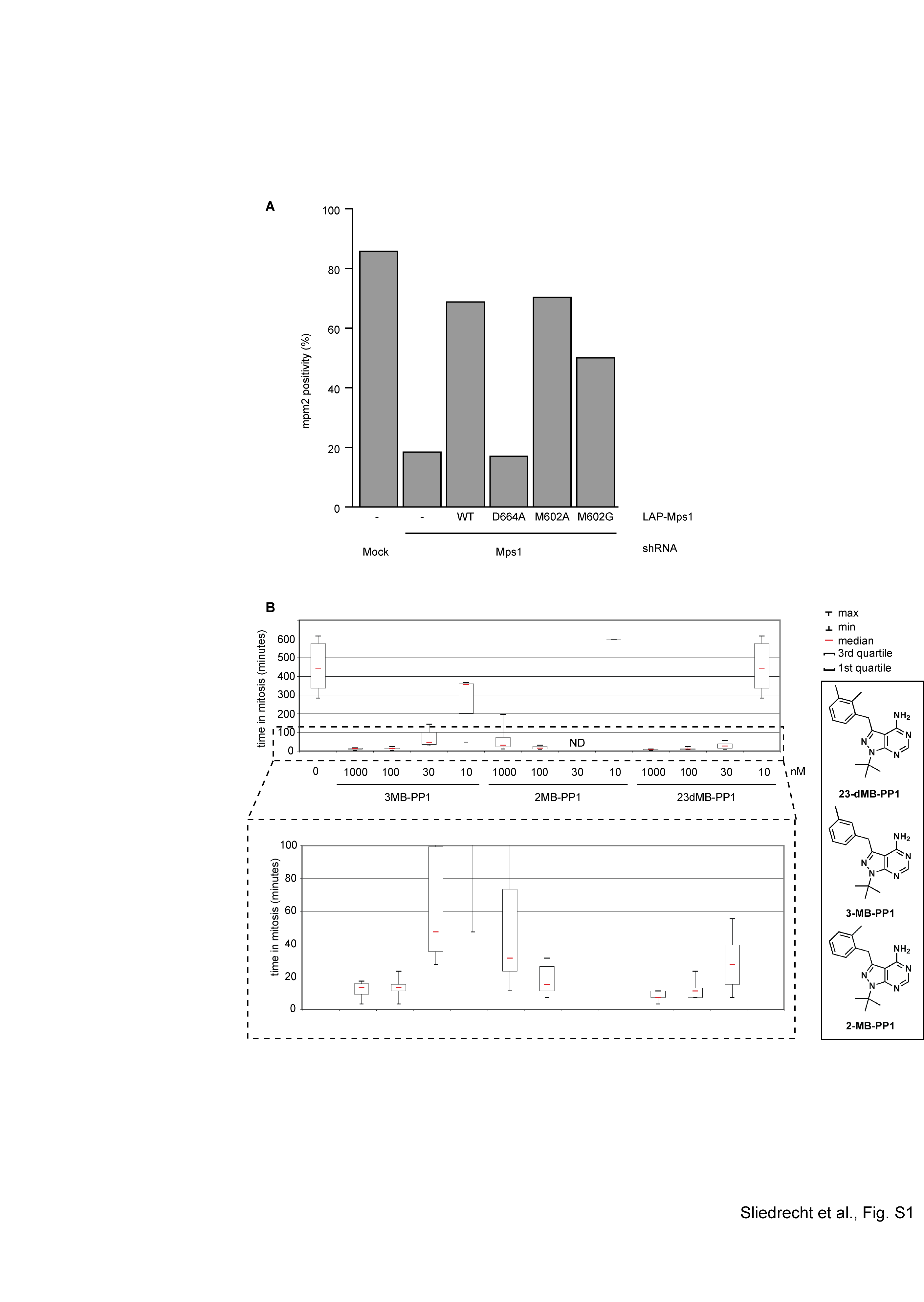

Supplement: Figure S1 — A: Quantification of flow cytometric analysis of fraction of mitotic cells treated with nocodazole for 18 hours. Cells were transiently transfected with indicated LAP-Mps1 constructs. D664A is an enzymatically inactive mutant. B: Box-and-whisker diagrams of time spent in mitosis of UTR-Mps1as cell treated with taxol in combination with increasing concentrations of different bulky PP1 inhibitors. ND; not determined. (Molecular structures displayed in right panel.) (0.27 MB TIF) [file pone.0010251.s001.tif]

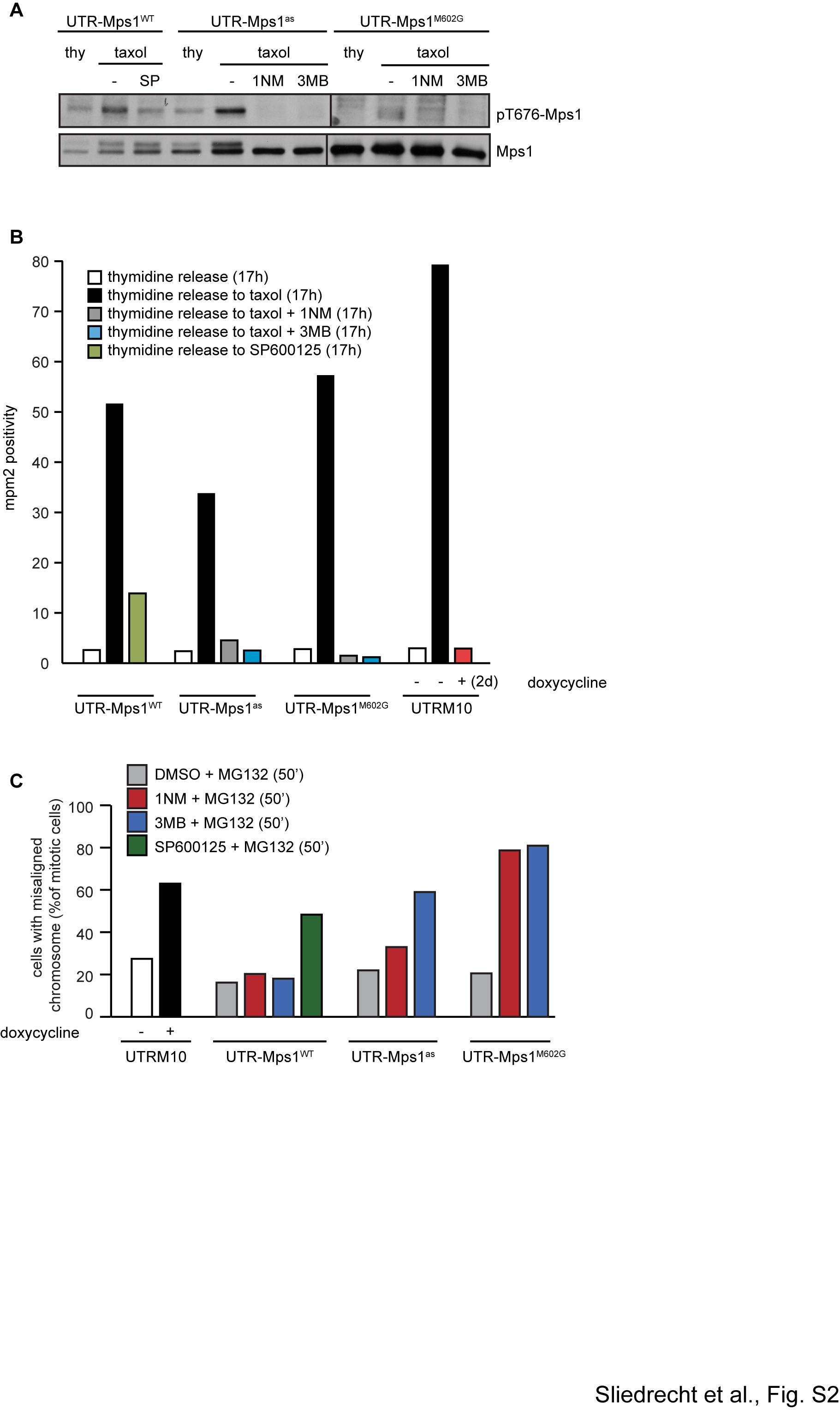

Supplement: Figure S2 — A: pT676-Mps1 and Mps1 immunoblot of UTR-Mps1WT, UTR-Mps1as and UTR-Mps1M602G cells treated with thymidine (thy) for 20 hours or with taxol and MG132 in combination with indicated inhibitors for 1 hour. 1NM, 1NM-PP1; 3MB, 3MB-PP1; SP, SP600125. B: Quantification of flow cytometric analysis of fraction of mitotic UTRM10, UTR-Mps1WT, UTR-Mps1as and UTR-Mps1M602G cells treated with taxol in combination with indicated inhibitors for 17 hours. C: Chromosome alignment in UTRM10, UTR-Mps1WT, UTR-Mps1as and UTR-Mps1M602G cells treated with MG132 for 50 minutes in combination with the indicated inhibitors. Graph displays percentages of cells with misaligned chromosomes. (0.84 MB TIF) [file pone.0010251.s002.tif]

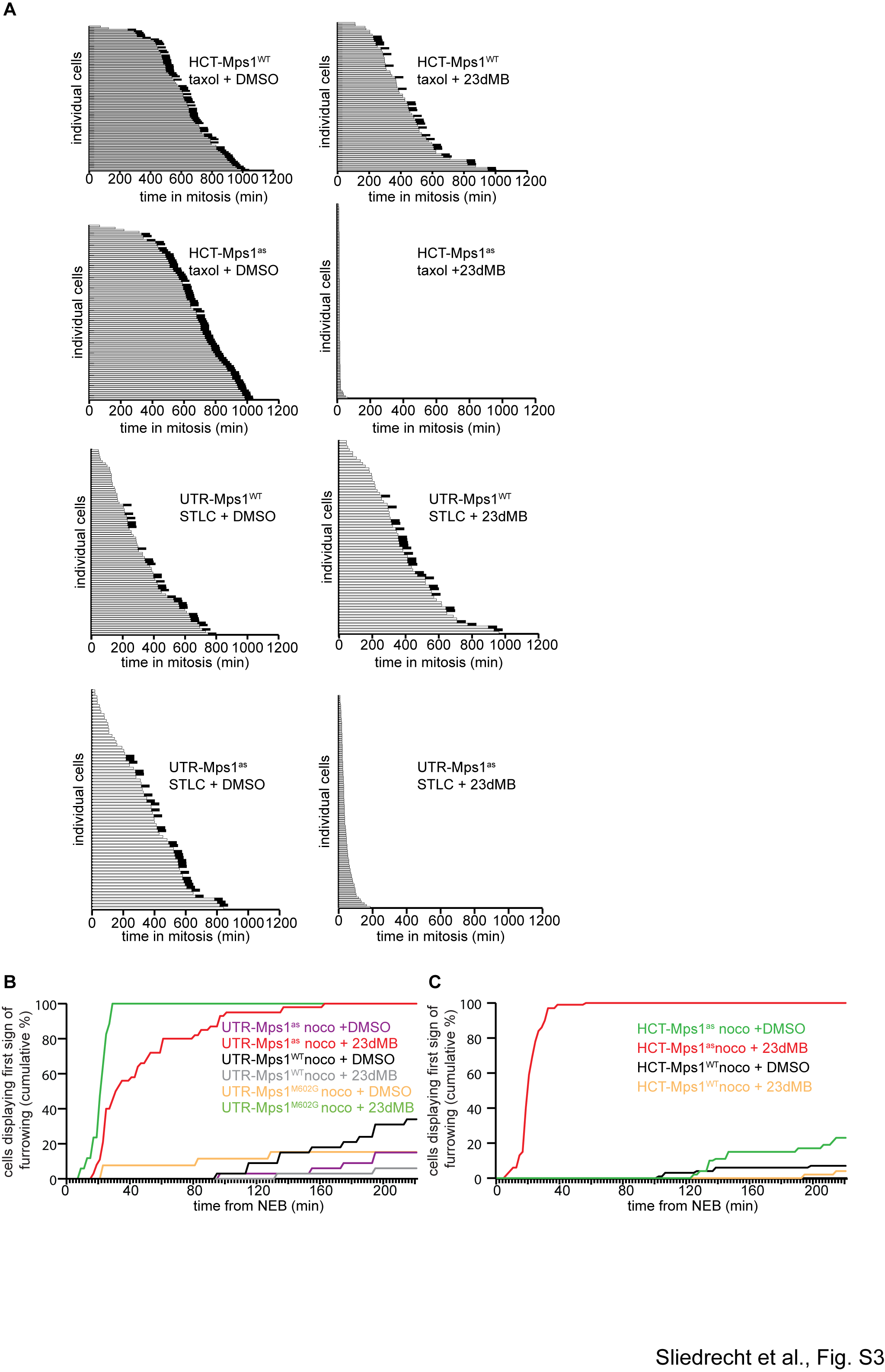

Supplement: Figure S3 — A–C: Time-lapse analysis by DIC microscopy of HCT- and UTR-derived cells treated with (A) taxol (HCT-derived cells) or STLC (UTR-derived cells) in combination with DMSO or 23dMB-PP1, or nocodazole (B, C) in combination with DMSO or 23dMB-PP1. Graphs in A display the time from NEB to the first signs of furrow ingression for individual cells. Black bars indicate end of time-lapse acquisition. Line graphs in B and C show cumulative percentages of UTR-derived cells (B) and HCT-derived cells (C) displaying first signs of furrowing at indicated times after NEB. (0.79 MB TIF) [file pone.0010251.s003.tif]

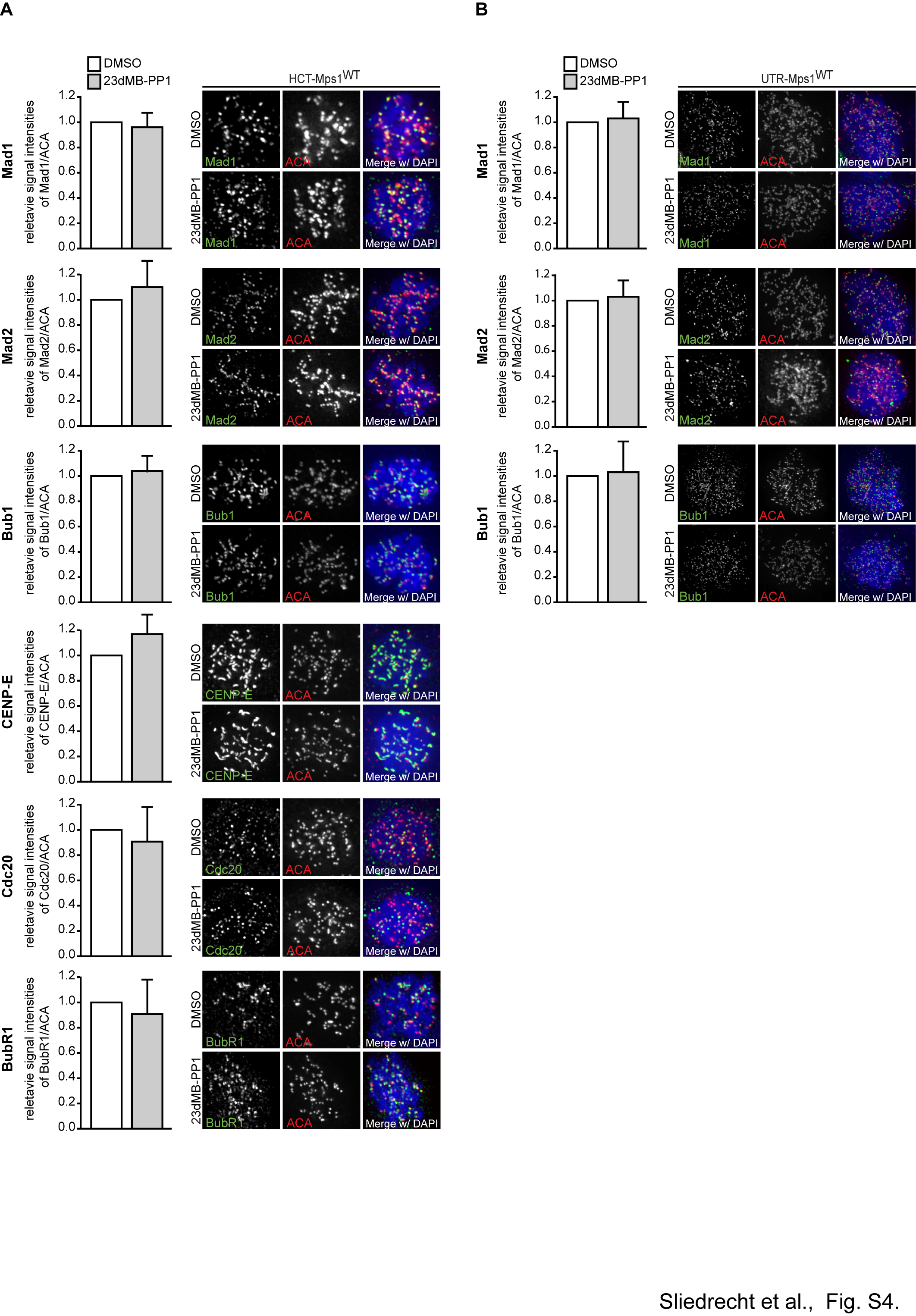

Supplement: Figure S4 — Immunolocalization of Mad1, Mad2, Bub1, BubR1, Cdc20 and CENP-E and centromeres (ACA) in HCT-Mps1WT (A) and UTR-Mps1WT (B) cells treated with nocodazole and MG132 for 1 hour in combination with DMSO or 23dMB-PP1. Graphs represent quantifications of kinetochore signal intensities for the investigated proteins at all kinetochores in a single cell as a ratio of the ACA signal. Data are average of three experiments, 10 cells per experiment (±SD). Representative images are shown. DNA (DAPI) is in blue. (3.76 MB TIF) [file pone.0010251.s004.tif]

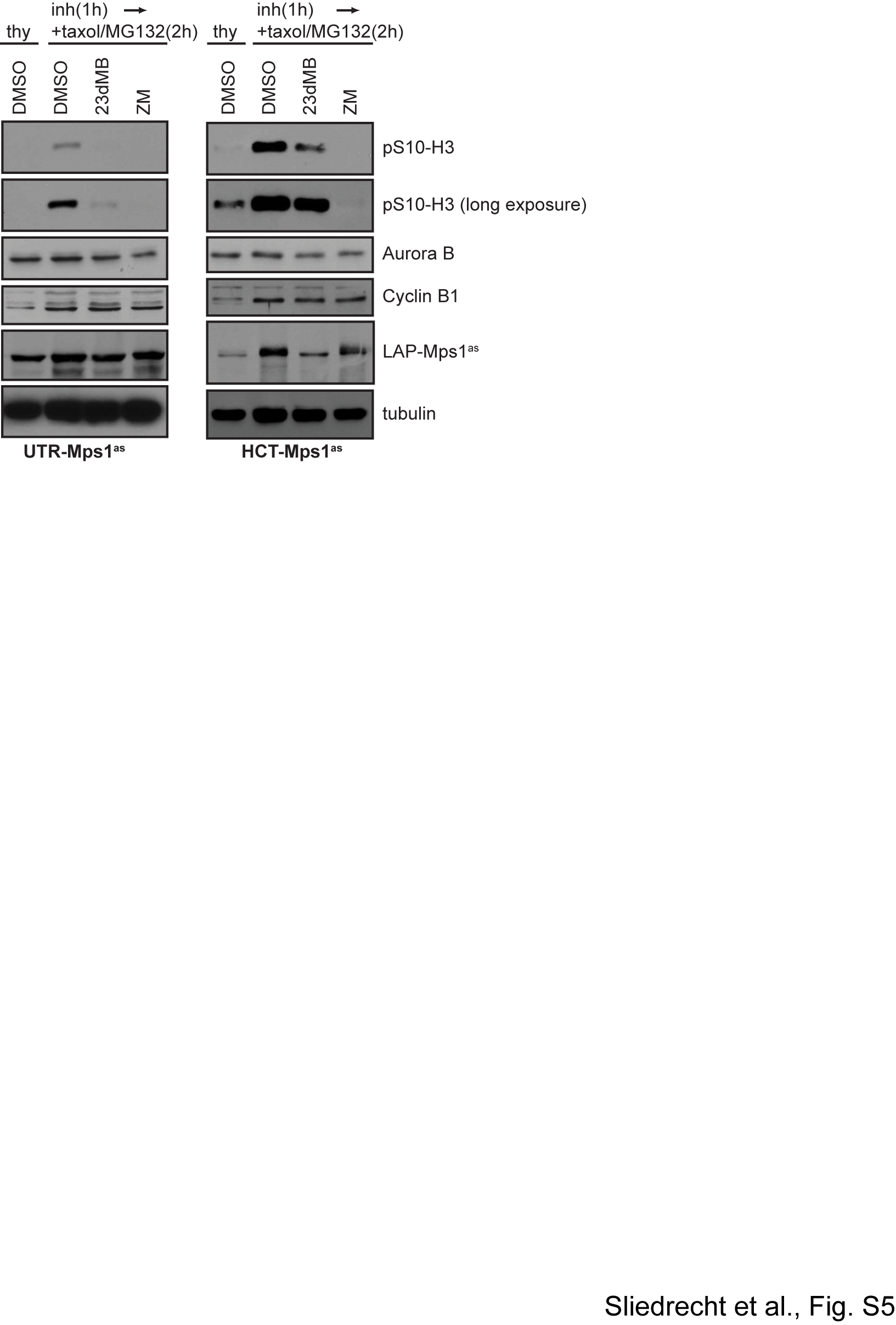

Supplement: Figure S5 — Immunoblots of the indicated proteins in lysates of the HCT- and UTR-Mps1 variant cell lines. Cells were treated with thymidine (thy), or with the indicated inhibitors for 1 hour followed by taxol/MG132 treatment for an additional 2 hrs. ZM, ZM447439. (0.71 MB TIF) [file pone.0010251.s005.tif]
